# Effects of BDNF and PEC Nanoparticles on Osteocytes

**DOI:** 10.3390/molecules25184151

**Published:** 2020-09-10

**Authors:** Thomas Leonhard Loy, David Vehlow, Vivien Kauschke, Martin Müller, Christian Heiss, Katrin Susanne Lips

**Affiliations:** 1 Experimental Trauma Surgery, Justus-Liebig-University, 35392 Giessen, Germany; thomasloy@hotmail.de (T.L.L.); Vivien.Kauschke@chiru.med.uni-giessen.de (V.K.); Christian.Heiss@chiru.med.uni-giessen.de (C.H.); 2 Department Functional Colloidal Materials, Leibniz Institute of Polymer Research, 01069 Dresden, Germany; Vehlow@ipfdd.de (D.V.); mamuller@ipfdd.de (M.M.); 3 Department of Trauma, Hand and Reconstructive Surgery, University Hospital of Giessen-Marburg GmbH, Campus Giessen, 35392 Giessen, Germany

**Keywords:** neurotrophin, brain-derived neurotrophic factor, polyelectrolyte complex nanoparticles, MLO-Y4, osteocyte, dorsal root ganglia, proliferation, vitality

## Abstract

Bone substitute materials loaded with mediators that stimulate fracture healing are demanded in the clinical treatment in trauma surgery and orthopedics. Brain-derived neurotrophic factor (BDNF) enhances the proliferation and differentiation of mesenchymal stem cells into osteoblast. To load the implants with BDNF, a drug delivery system that allows the release of BDNF under spatiotemporal control would improve functionality. Polyelectrolyte complex nanoparticles (PECNP) have been reported as a suitable drug delivery system. The suitability of PECNP in contact with osteocytes as the main cell type of bone is not known so far. Thus, we aimed to verify that BDNF and PECNP loaded with BDNF (PECNP+BDNF) as well as pure PECNP have no negative effects on osteocytes in vitro. Therefore, the murine osteocyte cell line MLO-Y4 was treated with BDNF and PECNP+BDNF. The effects on proliferation were analyzed by the BrdU test (*n* = 5). The results demonstrated a significant increase in proliferation 24 h after BDNF application, whereas PECNP+BDNF did not lead to significant changes. Thus, we conclude that BDNF is an appropriate mediator to stimulate osteocytes. Since the addition of PECNP did not affect the viability of osteocytes, we conclude that PECNP are a suitable drug delivery system for bone implants.

## 1. Introduction

The most abundant cells in the skeleton are osteocytes embedded in bone matrix. They are able to sense the mechanical load, orchestrate bone remodeling, and regulate bone formation [1]. Osteocytes communicate with osteoblasts and osteoclasts via cell contacts through dendritic processes or soluble factors that are secreted into the pericellular space and distributed through the lacunocanalicular network [2]. Mature osteocytes synthesize the protein sclerostin (gene name: SOST), which is an important negative regulator of bone formation by osteoblasts [1]. Receptor activator of nuclear factor-κB ligand (RANKL) also contributes to the secretome of osteocytes and enhances the differentiation of osteoclasts by binding to receptor activator of nuclear factor-κB (RANK) expressed at the surface of osteoclasts [3,4]. The decoy receptor osteoprotegerin (OPG) is able to block the binding site of RANKL and therefore can inhibit the stimulation of osteoclasts by RANKL [3]. OPG is synthesized by osteocytes as well as by osteoblasts.

Brain-derived neurotrophic factor (BDNF) is a well-known growth factor in the nervous system [5]. Yamashiro et al. 2001 detected BDNF and TrκB mRNAs as well as BDNF protein in rat osteoblasts at the site of intramembranous bone formation during development [6]. During fracture healing, BDNF and TrkB were upregulated compared to adult physiological bone as shown in mice [7] and humans [8]. In vitro application of BDNF resulted in enhanced proliferation and differentiation of MSC during their fate into osteoblasts [9]. Stimulation of proliferation by application of exogenous growth factors is controversially discussed since Carragee et al. reported concerns regarding tumor formation in association with the usage of the growth factor bone morphogenetic protein 2 (BMP2) in spine surgery [10]. BDNF is also expressed in tumors, e.g., osteosarcoma [11], and stimulates cancer growth and formation of metastasis through binding to TrκB as shown for breast cancer [12]. Thus, BDNF might also hold the risk of increasing cancer and other off target lateral effects. Thus, its effect on cells needs to be analyzed in detail before usage in vivo. In patients with multiple myeloma BDNF stimulated bone destruction by promoting osteoclastogenesis, while the inhibition of the BDNF/TrkB axis blocked osteoclastogenesis and bone destruction [13,14,15]. The addition of BDNF to monocyte cultures during differentiation into osteoclasts did neither impair the osteoclastogenesis nor the activity of osteoclasts if they were harvested from healthy donors [16,17]. In non-tumorigenic animal models, e.g., in murine and rat fracture models, the application of BDNF revealed enhanced bone formation and fracture healing [18,19]. The positive effect of BDNF on fracture healing was also determined when BDNF was released from a bone substitute material [18]. To refine the release of BDNF by bone substitute materials like calcium phosphate cements, it would be promising to couple BDNF to a drug delivery system. 

Polyelectrolyte complex nanoparticles (PECNP) have been described recently as a drug-delivery system. PECNP are generated by mixing anionic (n−) cellulose sulfate (CS) and cationic (n+) polypeptide poly (l)-lysine [20]. They feature wet-adhesive properties as biomaterials, loadability and low initial burst, as well as sustained and retarded release for a variety of drugs [20]. PECNP show good cytocompatibility with human mesenchymal stem cells (MSC) in vitro [20]. Their application to osteoclasts also resulted in good cytocompatibility at low concentrations, whereas higher concentrations reduced the formation and activity of osteoclasts [17]. To our knowledge, the cytocompatibility of PECNP with osteocytes has not been investigated so far.

In the present study, we aimed to investigate the effect of PECNP on proliferation of osteocytes as well as the release of BDNF by PECNP. The main objective of our study is to shed light on the function of BDNF in osteocyte proliferation. We asked whether the application of exogenous BDNF alters the endogenous BDNF production by cells of the peripheral nervous system. Therefore, we established a coculture model of the murine osteocytic cell line MLO-Y4 [21] with primary murine dorsal root ganglia (DRG) as endogenous BDNF source in accordance with the coculture system of Boggs et al. 2011 [22]. Then, BDNF, PECNP, and PECNP loaded with BDNF were added and the proliferation and gene expression of the MLO-Y4 osteocytes were analyzed.

## 2. Results

### 2.1. Selection of the PECNP+BDNF Loading

**Figure 1:**
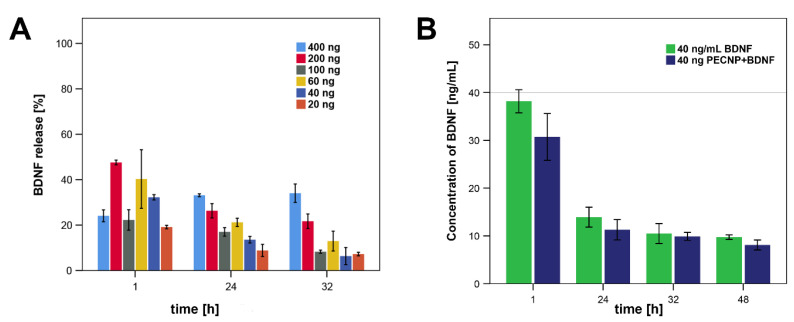
BDNF ELISA. (**A**) Amount of BDNF measured by ELISA in correlation to the calculated maximal amount that can be released by the different samples of PECNP+BDNF with 20, 40, 60, 100, 200, and 400 ng BDNF after 1, 24, and 32 h. (**B**) The release kinetics of PECNP+BDNF containing initial amount of 40 ng BDNF was most similar to the concentration of 40 ng/mL pure BDNF.

Since it was not possible to increase the concentration of BDNF that was loaded to PECNP, it was decided to increase the amount of PECNP added to the cell cultures. Here, we determined the BDNF release at time points 1, 24 and 32 h for PECNP+BDNF dispersions with a BDNF amount of 20, 40, 60, 100, 200 and 400 ng (*n* = 2, Figure 1A). Interestingly, with the exception of the sample 400 ng PECNP+BDNF, all PECNP+BDNF samples showed an initial burst at 1 h, and afterwards a reduced release rate. The 400 ng loaded PECNP+BDNF demonstrated an increased liberation after 24 and 32 h, compared to 1 h. The 40 ng loaded PECNP+BDNF exhibited the most similar release rate compared to the addition of sole BDNF at a concentration of 40 ng/mL (Figure 1B). Thus, we decided to use 40 ng loaded PECNP+BDNF for the subsequent analysis of proliferation. 

### 2.2. Proliferation

**Figure 2:**
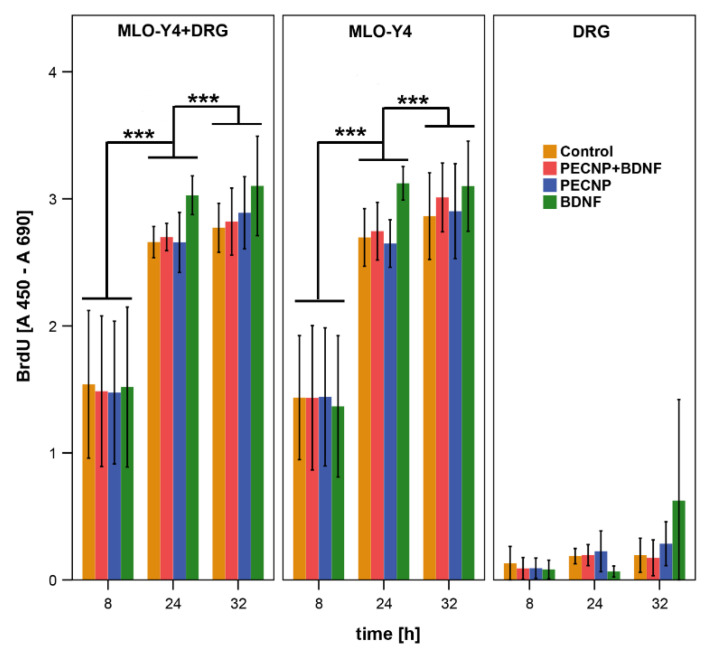
Proliferation measured by BrdU increase in cocultures of MLO-Y4+DRG and in single cultures of MLO-Y4 from 8 to 24 h and from 8 to 32 h of incubation time of BDNF, PECNP, PECNP+BDNF, as well as without supplements. No significant changes in proliferation were measured in pure DRG cultures. The asterisks (***) indicate statistically significant differences, with a likelihood of *p* ≤ 0.001.

The proliferation of osteocyte-like cells of the cell line MLO-Y4 was analyzed by BrdU assay as described in Section 4.4. As shown in Figure 2, there was nearly no proliferation in DRG cultures whereas in MLO-Y4 as well as in cocultures the proliferation increased from 8 to 24 h. No significant increase was measured from 24 to 32 h. Since the DRG culture does not show significant proliferation, it can be suspected that the increased proliferation in the cocultures is caused by effects of the MLO-Y4 and not by DRGs.

**Figure 3:**
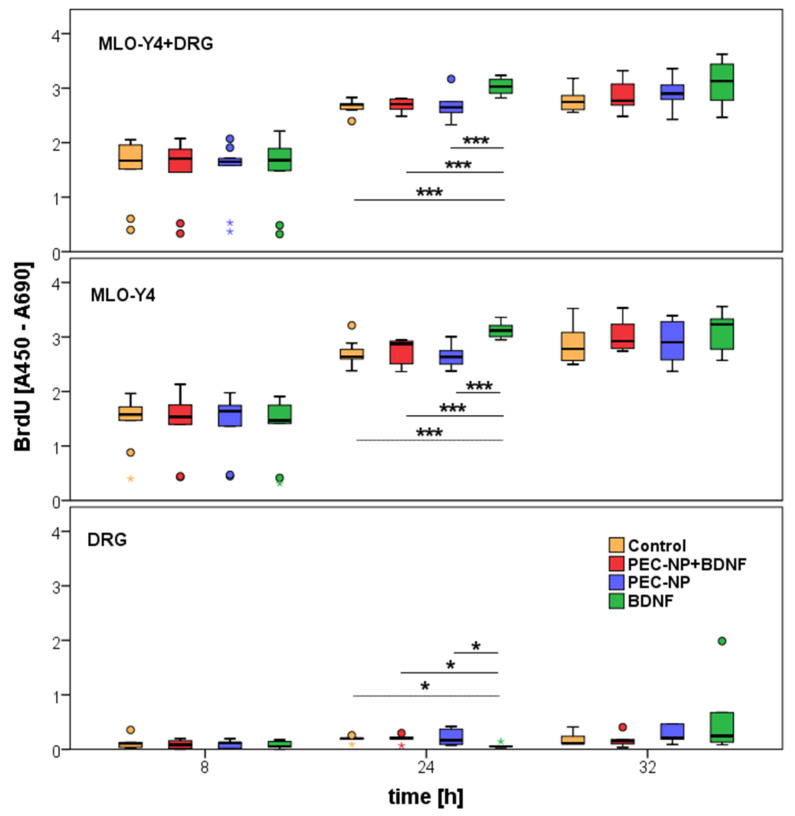
Proliferation increased in cocultures of MLO-Y4+DRG as well as in single culture of MLO-Y4 24 h after addition of BDNF, PECNP and PECNP+BDNF compared to cells without application of supplements whereas in cultures of pure DRG a reduction in proliferation was measured after application of pure BDNF. After incubation time of 8 and 32 h no significant differences in proliferation were measured by BrdU assay. One asterisk (*) indicates a statistically significant likelihood of *p* ≤ 0.05 and three asterisks (***) of *p* ≤ 0.001. Circles and colored asterisks indicate outliers.

In cocultures of MLO-Y4 and DRGs, we measured a significant increase 24 h after addition of BDNF (*p* < 0.001) compared to the application of pure PECNP, loaded PECNP+BDNF, as well as control cultures without any treatment (Figure 3). A similar result was obtained for MLO-Y4 cultured without DRG. The increase in the MLO-Y4 single cell cultures (15.8%) was more pronounced than in the cocultures (14%). In contrast, a significantly reduced proliferation rate was detected in the DRG cultures 24 h after addition of BDNF (*p* < 0.05). No significant changes were found after 8 and 32 h of incubation time (Figure 3).

The increase in proliferation in cultures incubated with BDNF was significantly higher than in cultures with PECNP+BDNF, PECNP and cultures without treatment (*p* < 0.001, Figure 3). Thus, dissolved BDNF in cell culture medium up-regulated proliferation more than PECNP+BDNF. No significant differences were measured between cell cultures treated with PECNP+BDNF and PECNP alone. Hence, the functionalization of PECNP or the release of BDNF from PECNP+BDNF was not effective enough. Comparison of the untreated cells with cells administrated with PECNP+BDNF or PECNP alone showed no significant changes in proliferation. Therefore, we assume that the PECNP did not have any positive or negative effects on the proliferation rate of MLO-Y4. In regard of proliferation rate, the PECNP showed good cytocompatibility with murine osteocytes.

### 2.3. Real-Time Reverse Transcriptase Polymerase Chain Reaction (Real-Time RT-PCR)

**Figure 4:**
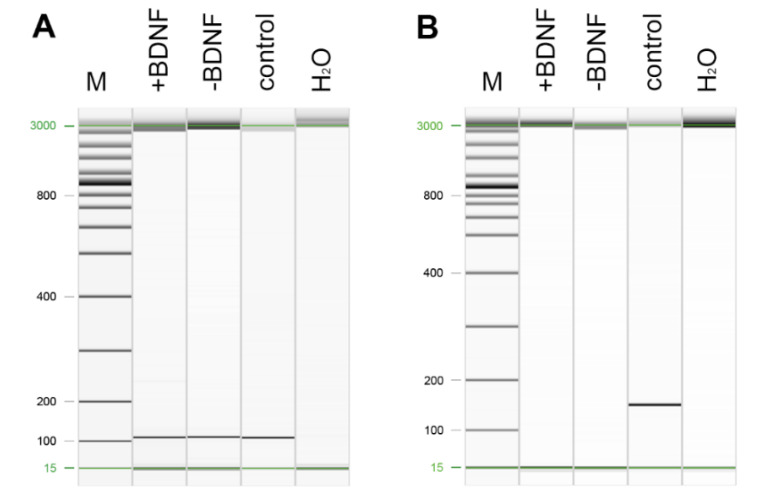
RT-PCR of TrkB (**A**) and SOST (**B**). TrkB PCR product was found in MLO-Y4 treated with and without BDNF as well as in the positive control (murine brain) at the appropriate height (**A**). SOST was not detected in MLO-Y4 (**B**). Messenger RNA of murine bone was used as positive control where a PCR product of appropriate height was found. M standard marker [bp]. H_2_O control run without cDNA template.

**Figure 5:**
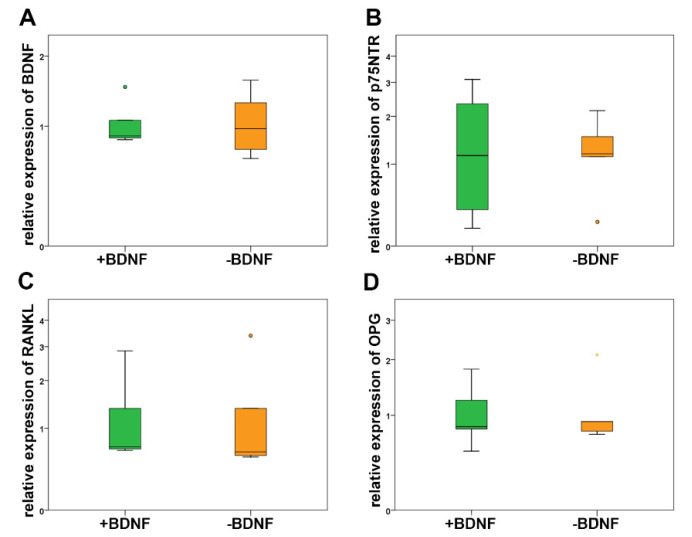
Real-time RT-PCR did not show significant differences in the relative expression of BDNF (**A**), p75NTR (**B**), RANKL (**C**), and OPG (**D**) in MLO-Y4 treated with BDNF compared to application without BDNF. Circles and colored asterisks indicate outliers.

To learn more about the down-stream signaling pathway of BDNF in osteocytes and the regulation of mRNA expression after addition of exogenous BDNF, we performed real-time RT-PCR. Our results showed an expression of mRNA of BDNF, its specific receptor TrkB as well as the more common receptor p75NTR in MLO-Y4 with and without administration of BDNF (Figure 4). No significant differences were measured in BDNF and p75NTR mRNA expression in MLO-Y4 treated with BDNF compared to plain MLO-Y4 (Figure 5). The expression of TrkB mRNA was near the detection level of real-time RT-PCR and therefore, it was not possible to calculate the relative expression of TrkB. However, the exogenous application of BDNF did not alter the endogenous synthesis of BDNF in MLO-Y4. The increased availability of BDNF after exogenous application did not lead to a down-regulation of TrkB and p75NTR in a kind of negative feedback mechanism.

Since SOST and the RANKL-OPG-axis are important mechanisms of osteocytes that are involved in regulation of the activity of osteoblasts and osteoclasts, we analyzed their mRNA expression in MLO-Y4 after application of BDNF. We did not detect the expression of SOST mRNA, whereas RANKL and OPG mRNAs were expressed in samples with and without BDNF treatment (Figure 5). However, RANKL and OPG mRNA expression was not regulated after addition of BDNF (Figure 5). Exogenous BDNF was not involved in the regulation of osteoblasts via SOST. In addition, BDNF application did not facilitate the osteoclastogenesis and activity of osteoclasts by RANKL and its decoy receptor OPG.

## 3. Discussion

Our results demonstrated for the first time that BDNF increased the proliferation of MLO-Y4 in vitro. The MLO-Y4 is a murine osteocyte cell line that was established and characterized by Kato et al. [21]. The state of the art suggests that primary osteocytes do not proliferate in vivo. During generation, the osteocyte cell line was modified regarding its proliferation rate. MLO-Y4 is able to proliferate as reported previously [23], but still holds the main characteristics of osteocytes [21]. Recent studies of our group have shown that the concentration of 40 ng/mL BDNF in the cell culture medium stimulated alkaline phosphatase (ALP) activity of osteoblasts and enhanced the cell number of MSC as progenitors of osteoblasts [9], whereas no alteration in the activity of osteoclasts and their differentiation was found [16,17]. Studies of other groups have reported positive effects with other concentrations ranging from 5 ng/mL to 100 ng/mL BDNF, and several cell types of different species to be affected when analyzed by a panel of functional assays [24]. Only a small number of them described positive effects on proliferation. Li et al. measured an increase in proliferation of rat neuronal stem cells with 40 ng/mL BDNF [25]. A serial increase in proliferation was reported in cells of human periodontal ligament treated with BDNF concentrations ranging from 10 to 100 ng/mL [26]. Liu et al. verified the positive effect of 100 ng/mL BDNF on proliferation of human MSC [27]. In the murine osteoblast cell line MC3T3 proliferation was not enhanced by using 80 ng/mL of BDNF [19]. Thus, the appropriate concentration of BDNF for stimulation of proliferation is controversially discussed, and might be impaired by species and cell types. We decided to use 40 ng/mL BDNF, which, in our hands, showed positive effects on human MSC and osteoblast and did not stimulate osteoclasts. 

BDNF is involved in manifold systemic metabolic processes of the body, and therefore a relatively high concentration of 32.69 ± 8.33 ng/mL BDNF is present in human blood serum [28] and down-regulated during several diseases, e.g., systemic lupus erythematosus [29] and schizophrenia [30]. Thus, we suppose that the systemic application of BDNF would lead to severe side effects. A possible way to avoid this is the loading of BDNF to a drug delivery system that releases the drug only at the place of interest but not systemically. Müller et al. (2018) described that PECNP are a suitable system for loading of drugs and functionalization of bone substitute materials and implants [31]. Of course, the drug delivery system must be biocompatible. The cytocompatibility of PECNP in human MSC has been proven in previous studies [20,32]. Only osteoclasts showed a reduced activity and delayed differentiation after addition of high concentrations of PECNP [17]. Here, we measured no significant changes in proliferation of MLO-Y4 after addition of PECNP compared to the controls without any treatment. Thus, this is a first hint for the cytocompatibility of PECNP regarding osteocytes. After carefully investigating all other aspects of in vitro cytocompatibility (e.g., viability assays), it will be necessary to verify the biocompatibility of PECNP in an appropriate animal model in vivo.

Drug delivery systems have the advantage that they release drugs under control of time and concentration. Most suitable would be a liberation without initial burst but with a long duration of an appropriate concentration of the drug. To receive a similar BDNF concentration in cell culture medium as with the pure BDNF we tested several concentrations. Since up to now, it is not possible to increase the BDNF concentration that is loaded to PECNP we added equivalent more or less PECNP into the medium and investigated then the BDNF concentration by enzyme-linked immunosorbent assay (ELISA). Regarding the initial burst, the 400 ng loaded PECNP+BDNF are most promising. However, the released BDNF concentration was 132.3 ng/mL and 136.2 ng/mL at 24 h and 32 h for the 400 ng PECNP+BDNF. This is above the concentrations of BDNF that have been tested thus far. Application of 40 ng/mL pure BDNF into the cell culture medium resulted in a decline of BDNF concentration measured by ELISA as shown in Figure 1B. Because of the similar graph resulting after application of 40 ng PECNP+BDNF, we decided to use the 40 ng loaded PECNP+BDNF for further investigations. However, the commercial BDNF ELISA kit is not able to distinguish between free BDNF and PECNP+BDNF. Therefore, we added a filtration step, where we tried to eliminate the PECNP+BDNF. Nevertheless, this might be a limitation in our measurements with respect to BDNF release by PECNP+BDNF.

No relevant differences in proliferation were determined in MLO-Y4+DRG compared to single cultures of MLO-Y4 by means of BrdU assay. Thus, the cells of the DRG cultures did not alter the viability of MLO-Y4 and their proliferation rate. Our cultures of DRG contained neurons as well as Schwann cells and some remaining fibroblasts. As described by Boggs et al. (2011) we treated the isolated DRG cells with arabinosyl cytosine (ARAC) to inhibit the proliferation of cells with a short cell cycle like fibroblasts [22]. Before adding the MLO-Y4, cultures were carefully washed to remove ARAC completely. DRG neurons as well as Schwann cells are able to synthesize and secrete BDNF. The exogenously added BDNF was mature BDNF. Mature BDNF binds to TrkB and acts as survival factor and promotor of cellular functions. Endogenously released BDNF contains pro as well as mature BDNF. Pro BDNF usually binds to the more common p75NTR receptor and induces apoptosis. Thus, it is important to learn whether application of exogenous BDNF modifies the release rate of endogenous BDNF as well as their composition of pro and mature BDNF. Since the single cultures of DRG demonstrated a reduced proliferation rate after 24 h when treated with BDNF, a negative effect of the exogenous BDNF application on possible endogenous BDNF sources cannot be completely excluded. However, this effect was small, and was only observed at 24 h, but not at 8 and 32 h of incubation time. Remarkably, the application of PECNP and PECNP+BDNF did not reduce the proliferation rate. Thus, PECNP are also cytocompatible for DRG neurons. On the other hand, it has to be considered that BDNF should not be applied systemically to avoid side effects on the nervous system. In addition, it can be implied that the DRG neurons are especially sensitive in single culture with a low cellular density and that the addition of other cells, e.g., MLO-Y4 might increase the viability of DRG neurons by making contact with them. However, at the moment, these are only speculations that have to be analyzed in detail by future investigations. 

To our knowledge, this is the first study that has detected mRNA expression of BDNF as well as its specific receptor, TrkB, and its more common receptor, p75NTR, in osteocytes. Often, mostly provocatively, the osteocyte network is compared with neuronal networks. The expression of typical neuronal markers by osteocytes might give breeding ground to compare osteocytes and neurons. Besides BDNF, MLO-Y4 also express other neurogenic markers, e.g., neuropeptide Y and reelin [23], that might be involved in non-neuronal regulation of bone metabolism.

Osteocytes synthesize very typical molecules that are important for regulation of bone remodeling, e.g., sclerostin (gene name SOST) as well as RANKL and OPG as two proteins of the RANKL/RANK/OPG axis, which is involved in the stimulation of osteoclastogenesis and osteoclast activity. In our study, we did not detect the expression of SOST. It is controversial as to whether MLO-Y4 express SOST [33,34] or not [35]. One reason for this controversy might be different cell culture conditions. It can also be suspected that SOST is not expressed by MLO-Y4 because MLO-Y4 are at a developmental stage of juvenile osteocytes, whereas SOST is typically expressed in developmentally mature osteocytes. SOST expression has the function to decrease bone formation by osteoblast. Maybe, MLO-Y4 did not get the appropriate stimulus to express SOST in our culture system because it was not necessary to decline the osteoblast functions. Still, MLO-Y4 expressed RANKL and its decoy receptor OPG in our cell cultures. Neither RANKL nor OPG were regulated by addition of BDNF. Thus, BDNF does not enhance bone resorption through secretion of RANKL by osteocytes. On the contrary, BDNF also doesn’t increase the expression of OPG to inhibit osteoclastogenesis and activity of osteoclasts. We suppose that BDNF does not affect osteoclasts in cell cultures of cells from healthy individuals as recently shown [16,17]. The situation in samples and cells of patients with multiple myeloma and myeloma animal models is different [13,15]. It might be interesting if the expression of RANKL and OPG is regulated additionally in cocultures of MLO-Y4 and multiple myeloma above the already shown regulation of RANKL in myeloma-osteoblasts [14]. 

## 4. Materials and Methods

### 4.1. PEC-NP

As a drug delivery system, we used polyelectrolyte complex nanoparticles (PECNP), which have previously been described by Vehlow et al. (2016) [20]. In brief, they were produced by first mixing low (CS-0.5, Euroferm, Erlangen, Germany) and high anionic (n−) cellulose sulfate (CS-3.0, 1,200,000 g/mol, Janssen Chimica, Belgium), which corresponds to CS with an average substitution degree of 1.0 (CS-1.0). CS-1.0 was then mixed with 0.002 M cationic (n+) polypeptide poly (l)-lysine (PLL, 30,000–70,000 g/mol, Sigma-Aldrich, St. Louis, MO, USA) and a molar charge ratio of n−/n+ = 1.1 was reached. For the cell culture experiments with unloaded PECNP, the concentration of PECNP in the cell culture medium was always 6.4 nM. For those with BDNF loaded PECNP at first a dispersion of PECNP was generated, to which BDNF was dosed so that the PECNP+BDNF dispersion contained 0.0083 µg/µL of BDNF. Then, this PECNP+BDNF dispersion was dosed in various amounts.

### 4.2. Cell Culture

The usage of mice for generation of cell cultures was approved by the animal care officer of the Justus-Liebig-University Giessen (AZ 508_M). Adult C57BL/6 mice (*n* = 5) were sacrificed. DRGs were extracted and incubated for 30 min at 37 °C in a solution of 1 mg/mL collagenase type I (125 collagen digestion unit (CDU)/mg; Sigma, Steinheim, Germany) and 1 mg/mL dispase II (≥0.8 U/mg; Roche), in Hibernate-A medium (Roche, Mannheim, Germany) containing 1% N1 medium supplement (Roche), 100 units/mL penicillin (Life Technologies, Darmstadt, Germany), 0.1 mg/mL streptomycin (Life Technologies), and 5% fetal bovine serum (FBS, Biochrom, Berlin, Germany). The DRG homogenate was filtered through a cell strainer with a pore size of 100 µm. The flow through was collected in 4 mL Hibernate-A medium and centrifuged for 3 min at 200 g. The pellet was washed carefully with Hibernate-A medium, suspended in 2 mL Neurobasal medium (Life Technologies) containing 10 nM arabinosyl cytosine (ARAC, Sigma) and seeded with a density of 500 cells/cm^2^ onto a 96 well plate coated with 15.6 µg/cm^2^ poly (l)-lysine (Specialty Media, Darmstadt, Germany) and 1.6 mg/cm^2^ laminin (Specialty Media). After 24 h (h) medium was removed, attached cells were washed with PBS and cultured in Neurobasal medium without addition of ARAC. For cocultures, MLO-Y4 were seeded with a density of 5000 cells/cm^2^ to the DRG culture and cultured in Dulbecco’s Modified Eagle Medium (DMEM)-F12 medium (Life technologies) with 1% N1 supplements, 0.2% gentamicine/amphotericine and 5% FBS. The MLO-Y4 purchased from LGC Standards GmbH (ATCC, Wesel, Germany) were originally generated and described by Kato et al. (1997) [21]. The cells were regularly controlled and photographed during cell culture incubation by light microscopy using an inverse microscope (Axiovert 10, Zeiss, Oberkochen, Germany) equipped with a Stingray F-145 camera (Allied Vesion Technologies, GmbH, Stadtroda, Germany).

### 4.3. BrdU Assay

Proliferation was measured by the colorimetric 5-bromo-2′desoxyuridine (BrdU) assay (Roche) as described previously [36]. In brief, 10 µM BrdU and the supplements (40 ng/mL BDNF; 0.6% (*V*/*V*) of PECNP+BDNF, or 0.4% (*V*/*V*) of PECNP per well) were added to cell culture medium and incubated for 8, 24 and 32 h. Then, cells were fixed with 200 µL FixDenat (kit component) for 30 min at room temperature and peroxidase conjugated BrdU antibody (kit component) was added followed by substrate solution (kit component). The reaction was stopped with 25 µL of H_2_SO_4−_ and the absorption was measured at 450 nm with a reference at 690 nm using the plate reader (Synergy HT, BioTek, Winooski, VT, USA). The assay was repeated 5 times (*n* = 5). Every condition was measured as duplicate and the mean was used for further calculations.

### 4.4. BDNF Enzyme-Linked Immunosorbent Assay (ELISA)

An ELISA (Quantikine ELISA Human Free BDNF, R&D Systems, Abingdon, UK) was used for determination of BDNF concentrations in medium according to the manufacturer’s protocol. In brief, sterile filtered samples of PECNP+BDNF with initial BDNF amounts of 20, 40, 60, 100, 200 and 400 ng BDNF and sole BDNF (40 ng/mL) were diluted in medium. After an incubation time of 1, 24, 32 and 48 h, an aliquot of 10 µL was taken, diluted with RD1S diluent (kit component) and pipetted to the ELISA well plate. After 2 h incubation in the dark, 100 µL detection antibody was applied and incubated for 1 h. After removal of the supernatant and several washing steps, 200 µL substrate (kit component) were added and incubated for 30 min in the dark. The reaction was stopped by addition of 50 µL stop solution (kit component) and measured with the plate reader at 450 nm with a reference at 540 nm. Duplicates were performed under all conditions, and their average was used for further calculations as well as a dilution series.

### 4.5. Real-Time Reverse Transcriptase (RT)-Polymerase Chain Reaction (PCR)

Total RNA of MLO-Y4 with and without BDNF treatment for 24 h (*n* = 5) was isolated with the RNeasy Mini Kit (Qiagen, Hilden, Germany) according to the manufacturer’s protocol. Then, the RNA was reverse transcribed with the Quantitect Kit (Qiagen), where first the contaminating DNA was removed by incubation with 2 µL DNA Wipeout buffer for 2 min at 42 °C. Afterwards, cDNA was diluted 1:4 with RNase-free water. For PCR, 4 µL diluted cDNA, 0.2 µL forward primer, 0.2 µL reverse primer (Table 1), and 0.6 µL RNase-free water were added to 5 µL Mastermix of the QuantiFast SYBR Green PCR Kit (Qiagen). The PCR was carried out in the LightCycler 2.0 (Roche) with the following conditions: 5 min denaturation at 95 °C, 40 cycles with 10 s denaturation at 95 °C and 30 s polymerization and elongation at 60 °C followed by melting curve analysis where the temperature increased stepwise from 60 to 95 °C. The melting curve proved the purity of PCR products. PCR products were additionally screened by gel electrophoresis using the QIAxcel Advanced System (Qiagen). Therefore, the samples were diluted 1:3 with DNA dilution buffer (Qiagen), attached to the QIAxcel Advanced System and compared with QX alignment marker (Qiagen). For semi-quantitative analyses, relative expression was calculated by the ΔΔ cycler threshold (Ct) method. βActin was used as reference gene.

### 4.6. Statistical Analysis

The results were analyzed statistically with the SPSS software (version 23.0, SPSS Institute Inc., Chicago, IL, USA), which was also used for the generation of graphs. First, the Kolmogorov-Smirnov Test was used to assess normality. Data from real-time RT-PCR were normally distributed and therefore they were subsequently analyzed by T-test. Since data from proliferation assays did not meet normality the Wilcoxon and Bonferroni tests were employed subsequently. Significance was determined by *p* ≤ 0.05. Data are given as box plots, where the bold line within the box indicates the median, small circles mark data beyond three standard deviations, and colored asterisks indicate extreme outliers. 

## 5. Conclusions

We conclude that BDNF stimulated the proliferation of cells of the murine osteocyte cell line MLO-Y4. Since previous studies demonstrated a positive effect on the differentiation of human MSC into osteoblasts by BDNF and no alterations in osteoclast differentiation and function, we suppose that BDNF is a suitable mediator for coating of implants and functionalization of bone substitute materials. Released BDNF from PECNP did not enhance the proliferation of MLO-Y4. PECNP showed good cytocompatibility with MLO-Y4. Thus, PECNP seem to be appropriate as drug delivery system. The release of functional BDNF by PECNP needs to be improved in future studies.

## Figures and Tables

**Table 1:** Mouse primers used for real-time RT-PCR analysis.

Primer	Sequence	Length [bp]	Accession No.
BDNF	for GACGACATCACTGGCTGACAC	100	NM_007540.4
	rev GTCCGCGTCCTTATGGTTTTC		
OPG	for ACTTCATCGAAAGCACCCTGT	181	NM_008764
	rev TGGTAGGAACAGCAAACCTGA		
p75NTR	for TGTGCGAGGACACTGAGC	98	NM_033217
	rev GGGCGTAGACCTTGTGATCC		
RANKL	for TCCTGTACTTTCGAGCGCAG	136	NM_011613
	rev TCAGGTAGTGTGTCTTCACTCTC		
SOST	for GCCTCCTCCTGAGAACAACC	143	NM_024449.6
	rev GGCCTGGGCCGTCTGTC		
TrkB	for ATCTCCGCTCACTTCATGGG	99	NM_001025074.1
	rev AATGTCAGTTGGCGTGGTC		
βActin	for TGTTACCAACTGGGACGACA	165	NM_0073933.3
	rev GGGGTGTTGAAGGTCTCAAA		

for: forward primer; rev: reverse primer.
